# New therapy by surfactant to avoid neuro/psychiatric problems caused by cerebral hypoxia

**DOI:** 10.1192/j.eurpsy.2021.1871

**Published:** 2021-08-13

**Authors:** L. Bracco

**Affiliations:** Scientific Director Of Lorenzo Bracco Foundation, Lorenzo Bracco Foundation, Torino, Italy

**Keywords:** Surfactant, Cerebral Hypoxia, COVID-19, Type II Alveolar Cells

## Abstract

**Introduction:**

Covid-19 causes neuro/psychiatric problems by cerebral hypoxia.

**Objectives:**

My therapy could allow us to cross Covid-19 infection by minimizing both immediate and chronic lung damage and would avoid many deaths and neuro/psychiatric problems from cerebral hypoxia.

**Methods:**

In the case of a Covid-19 lung infection, the virus infects type II alveolar cells which consequently reduce the production of pulmonary surfactant. The surfactant has the function of reducing the surface tension of the alveoli. The less pulmonary surfactant there is, the more the alveoli tend to collapse due to the increased surface tension of their surface. Consequently, the lung would tend to collapse, that is, to reduce its volume, but collapse is prevented by the muscular movement of inspiration, which instead increases its volume. This means that a “low-pressure area” is created in the interstitial space which attracts liquid and substances which are often inflammatory and which organize over time, giving rise to interstitial pneumonia.

**Results:**

I propose to administer the pulmonary surfactant to the patient Covid-19 in the presence of dyspnea and certainly during assisted ventilation. This technique is routinely used in preterm infants suffering from lack of pulmonary surfactant production due to the immaturity of type II alveolar cells, pending that once matured these cells produce it autonomously.

**Conclusions:**

Similarly, the administration of surfactant during Covid-19 lung infection would allow the correct amount of surfactant to be maintained during the acute phase of the infection and would give time for type II alveolar cells to heal and independently resume surfactant production.

**Disclosure:**

No significant relationships.

